# Growth Inhibitory Effects of Dipotassium Glycyrrhizinate in Glioblastoma Cell Lines by Targeting MicroRNAs Through the NF-κB Signaling Pathway

**DOI:** 10.3389/fncel.2019.00216

**Published:** 2019-05-28

**Authors:** Gabriel Alves Bonafé, Jéssica Silva dos Santos, Jussara Vaz Ziegler, Kazuo Umezawa, Marcelo Lima Ribeiro, Thalita Rocha, Manoela Marques Ortega

**Affiliations:** ^1^Laboratory of Cell and Molecular Tumor Biology and Bioactive Compounds, Post Graduate Program in Health Science, Universidade São Francisco (USF), Bragança Paulista, Brazil; ^2^Multidisciplinary Research Laboratory, Post Graduate Program in Health Science, Universidade São Francisco (USF), Bragança Paulista, Brazil; ^3^Department of Molecular Target Medicine, School of Medicine, Aichi Medical University, Nagakute, Japan; ^4^Clinical Pharmacology and Gastroenterology Unit, Post Graduate Program in Health Science, Universidade São Francisco (USF), Bragança Paulista, Brazil

**Keywords:** glioblastoma, nuclear factor kappa-B, dipotassium glycyrrhizinate, *miR16*, *miR146a*

## Abstract

It has been shown that nuclear factor kappa-B (NF-κB) is constitutively activated in glioblastoma (GBM), suggesting that the pathway could be a therapeutic target. Glycyrrhetic acid (GA), a compound isolated from licorice (*Glycyrrhiza glabra*), has been shown to decrease cell viability and increases apoptosis in human cancer cell lines by NF-κB signaling pathway suppression. Dipotassium glycyrrhizinate (DPG), a dipotassium salt of GA, has anti-inflammatory properties without toxicity. The current study examined the effectiveness of DPG as an anti-tumor in U87MG and T98G GBM cell lines. Additionally, we assessed DPG as a candidate for combinational therapy in GBM with temozolomide (TMZ). Our results demonstrated that the viability of U87MG and T98G cells significantly decreased in a time- and dose-dependent manner after DPG treatment, and the apoptotic ratio of DPG-treated groups was significantly higher than that of control groups. In addition, DPG in combination with TMZ revealed synergistic effects. Furthermore, the expression of NF-κB-luciferase-reporter in transfected GBM cell lines was remarkably reduced after DPG exposure by up-regulating *miR16* and *miR146a*, which down-regulate its target genes, *IRAK2* and *TRAF6*. A reduced neuro-sphere formation was also observed after DPG in both GBM cells. In conclusion, DPG presented anti-tumoral effect on GBM cell lines through a decrease on proliferation and an increase on apoptosis. In addition, our data also suggest that DPG anti-tumoral effect is related to NF-κB suppression, where *IRAK2*- and *TRAF6*-mediating *miR16* and *miR146a*, respectively, might be a potential therapeutic target of DPG.

## Introduction

Glioblastoma (GBM) represents 65% of all adult nervous system cancers, being the most common among astrocytic tumors and characterized with an average survival period of less than 15 months (Krex et al., 2007; Brandes et al., 2008; Johnson and O’Neill, 2012; Shahar et al., 2012). Current treatment options include surgery, radiation therapy (Shahar et al., 2012), and the use of temozolomide (TMZ), a radiosensitizing chemotherapeutic drug (Camphausen and Tofilon, 2007; Zhang and Gao, 2007; Taphoorn et al., 2015). The anti-tumor effect of TMZ is methylation of tumor cell DNA causing apoptosis cell death (Weller et al., 2005; Sengupta et al., 2012; Fan et al., 2013). However, the treatment options show a limited response and lead to drug resistance development (Zhang and Gao, 2007). Therefore, a better understanding of GBM molecular resistance to chemotherapy may contribute to the development of new therapeutic agents.

Many dietary supplements and plant-derived compounds have been published as promising anti-tumor products by enhancing apoptosis-related pathways in tumor cells (Cassileth and Deng, 2004; Cragg and Newman, 2005). Glycyrrhizin (G), a triterpene glycoside complex isolated from licorice, possesses anti-inflammatory and anti-tumor effect (Menegazzi et al., 2008). It has been shown that G is effective against colon, lung, leukemia, melanoma, and GBM cell lines (Abe et al., 1987; Chung et al., 2000; Kobayashi et al., 2002; Cassileth and Deng, 2004; Cragg and Newman, 2005; Menegazzi et al., 2008; Khan et al., 2013; Huang et al., 2014). In addition, the incidence of liver carcinogenesis in patients with hepatitis C was clinically reduced after G administration (Ikeda et al., 2006). GA, an aglycone of G, has been demonstrated to have apoptotic effects on human hepatoma, promyelocytic leukemia, stomach cancer, Kaposi sarcoma-associated herpesvirus-infected cells, and prostate cancer cells *in vitro* by inducing DNA fragmentation and oxidative stress (Hibasami et al., 2005, 2006; Sivasakthivel et al., 2008).

Both nuclear factor kappa B (NF-κB) and tumor necrosis factor-α (TNF-α) are the key factors involved in cancer-related inflammation (Mantovani et al., 2008). NF-κB mediates the transactivation of genes encoding inflammatory cytokines (e.g., TNF-α), anti-apoptotic factors (e.g., *BCL-2*), cyclooxygenase-2 (COX2), inducible nitric oxide synthase (iNOS), and angiogenic factors (e.g., *VEGF*) in inflammatory cells and on transformation by carcinogens (Orlowski and Baldwin, 2002; Mantovani et al., 2008). Interestingly, GA supplementation suppressed the development of precancerous lesions in the colon of rats and it reduced the infiltration of mast cells by suppressing Ki-67, NF-κB-p65, COX-2, iNOS, and VEGF and enhancing p53, connexin-43, caspase-9, and cleaved caspase-3 proteins. GA treatment significantly attenuated the level of TNF-α, and it reduced the depletion of the mucous layer as well as attenuated the shifting of sialomucin to sulfomucin (Khan et al., 2013). Since NF-κB has been previously demonstrated as constitutively activated in GBM, the expression of NF-κB-p65 in the nucleus was also remarkably reduced after GA treatment in the U251 GBM cell line (Li et al., 2014).

Both G and GA have been prescribed for several therapeutic purposes, as inflammation and cancer. However, side effects have pointed out the problem of their toxicity (Cosmetic Ingredient Review Expert Panel, 2007). Dipotassium glycyrrhizinate (DPG), a dipotassium salt of GA, has been recently used as a flavoring and skin conditioner agent with demonstrated anti-allergic and anti-inflammatory properties (Cosmetic Ingredient Review Expert Panel, 2007). It can inhibit the leukotriene and reduce histamine with an apparent lack of adverse side effects (Cosmetic Ingredient Review Expert Panel, 2007; Shim et al., 2012; Vitali et al., 2013). Recently, DPG has also been known as a detoxicant, with anti-ulcerative and anti-inflammatory effects (Shim et al., 2012; Vitali et al., 2013).

In the current study, we examined the effects of DPG on two different GBM cell lines. Antiproliferative responses of DPG on these cell lines and its potential to induce apoptosis were evaluated. Our findings suggest that DPG treatment can confer inhibitory effects on human GBM cell lines, including inhibiting proliferation and inducing apoptosis, which is possibly related to the NF-κB-mediated pathway through *miR16* and *miR146a* inhibition. Additionally, we suggested that DPG might be used for combinational therapy in GBM along with TMZ and we also provided information that brain tumor stem cells are targeted by DPG-mediating inhibition.

## Materials and Methods

### Reagents

Dulbecco’s Modified Eagle’s Medium (DMEM) high glucose and fetal calf serum (FCS) were obtained from Cultilab, Campinas, São Paulo, Brazil. DPG [chemical abstracts service (CAS) number 68797-35-3] and TMZ (CAS number 85622-93-1) were obtained from Verdi Cosméticos LTDA (Joanópolis, São Paulo, Brazil) and Sigma (Schering Plough Temodal^^®^^), respectively. Dehydroxymethylepoxyquinomicin (DHMEQ) was synthesized as previously described (Suzuki et al., 2004). It was dissolved in dimethyl sulfoxide (DMSO) (Synth, Diadema, São Paulo, Brazil) to prepare a 10 mg/ml stock solution. For single and combinatorial cell line treatments, TMZ was diluted in DMEM to prepare a 2,000 μM stock solution. All treatment assays were performed in the presence of 10% FCS.

### Cell Lines

U87MG and T98G cell lines were gently donated by Dr. Adriana da Silva Santos Duarte from Hemocenter, State University of Campinas, Campinas, São Paulo, Brazil. Both were cultured in DMEM supplemented with 10% FCS and 1% streptomycin/penicillin (Cultilab, Campinas, São Paulo, Brazil). For all experiments, 1 × 10^6^ cells/ml were seeded and grown for 48–72 h before experimental treatments. Cells were maintained at a 37°C, 5% CO_2_ environment and were passaged by Trypsin 0.25% (Cultilab) every 3–4 days. Cells were fed every 2–3 days and used for the experiments until the seventh passage after thawing.

### MTT Assay

Cell viability was determined by MTT assay using DPG concentrations based on a previous publication in a murine macrophage-like cell line, RAW264.7, a human intestinal colorectal adenocarcinoma cell line, Caco2, and human colon carcinoma cell line HT29 with 300 μM (Vitali et al., 2013). Briefly, cells were seeded in 96-well flat-bottom plates (0.2 × 10^6^ cells/plate), and 16 h later, cells were treated for different periods of time (24, 48, and 72 h) with different doses of DPG (100, 300, 500, 700, 1,200, 1,600, and 2,000 μM). Subsequently, cells were treated for different periods of time (24, 48, and 72 h) with higher doses of DPG: U87MG (2, 5, 8, 12, 15, 18, 20, 24, and 28 mM) and T98G (8, 12, 15, 18, 20, 24, 28, and 32 mM).

Considering the TMZ levels reported for GBM patients treated with standard therapy (Baker et al., 1999; Rosso et al., 2009; D’Alessandro et al., 2016; Pazhouhi et al., 2018), U87MG cells were treated using different doses of TMZ (25, 50, 75, 100, 125, 150, 175, and 200 μM) for 48, 72, and 168 h. Next, U87MG and T98G cells were treated using higher TMZ concentration (250, 500, 750, 1,000, 1,250, 1,500, 1,750, and 2,000 μM) for 48, 72, and 96 h. In addition, both cell lines were cultured in the presence of DPG (U87MG 18 mM; T98G 24 mM) in combination with low TMZ concentrations (25, 50, 75, 100, 125, 150, 175, and 200 μM) for 6, 12, 18, and 24 h and high TMZ concentrations (250, 500, 750, 1,000, 1,250, 1,500, 1,750, and 2,000 μM) for 48, 72, and 96 h. Untreated control cells were analyzed in all experiments, and all drug dose treatments were performed in triplicate.

After treatment, GBM cell lines were incubated with 0.2 μg/μl MTT (Sigma, St. Louis, MO, United States) for 4 h at 37°C. The formazan crystals were dissolved in 100 μl of DMSO and the absorbance was measured at 540 nm in a plate reader (Thermo Fisher, Waltham, MA, United States). Cell survival rates were expressed as percentages of the value of normal cells.

### Agarose Gel Electrophoresis Analysis for DNA Fragmentation

U87MG and T98G cells were cultured in 6-well tissue culture plates for 24 h, and subsequently, cells were exposed to DPG (U87MG: 18 mM; T98G: 24 mM) for 48 and 72 h. T98G was also treated using 24 mM DPG for 96 h. At the end of the period times, cells were suspended in 200 μl of PBS. DNA was isolated using phenol-chloroform-isoamyl alcohol DNA extraction (Barnett and Larson, 2012). The same amount of DNA samples was electrophoresed on a 1.5% agarose gel and visualized with ethidium bromide staining under UV illumination.

### Measurement of the Effect of DPG on Cell Viability *in vitro*

U87MG and T98G (0.4 × 10^6^ cells/well) were cultured in 24-well tissue culture plates in the presence of DPG (U87MG 18 mM; T98G 24 mM). Cells were washed in PBS and viable cell count was determined by trypan blue dye exclusion assay for 4 days. Untreated cells were used as a control, and the experiments were performed in triplicate.

### Determination of Apoptosis

U87MG and T98G cells were cultured in 96-well tissue culture plates for 24 h. Cell lines were then exposed to 18 mM for 72 h (U87MG) and 24 mM DPG for 96 h (T98G). Apoptosis was evaluated on cells after treatment with DPG by *in situ* terminal deoxynucleotidyltransferase-mediated dUTP-biotin nick end-labeling (TUNEL) assay, using the *in situ* cell death detection kit, fluorescein (Roche Applied Science, Mannheim, Germany) according to the manufacturer’s protocols. Apoptotic indexes were calculated by scoring four randomly selected fields and counting the number of apoptotic cells over the total of viable cells, which represented a quota in comparison with untreated cells. Cells were directly analyzed under a fluorescence microscope (Axio Vert. A1 ZEISS, Germany).

### Assessment of Apoptosis in U87MG and T98G Cells by Western Blot

The Western blot was performed following standard protocol to detect caspase-3 activating. Briefly, following incubation with DPG (U87MG: 18 mM; T98G: 24 mM) for 48 h, cells were lysed with ice-cold lysis buffer (EDTA 10 mM, Trizma base 100 mM, sodium pyrophosphate 10 mM, sodium fluoride 100 mM, sodium orthovanadate 10 mM, PMSF 2 mM, and aprotinin 1 mg/ml) for 30 min on ice. Then, collected proteins were subjected to SDS-PAGE gels and transferred onto PVDF membranes (Millipore, Burlington, MA, United States). Proteins were probed with specific primary antibodies and a rabbit polyclonal antibody to GAPDH used as a gel loading control (Santa Cruz Biotechnology, Dallas, TX, United States). Specific primary antibody against cleaved caspase-3 was purchased from Sigma, St. Louis, MO, United States. The secondary antibody (HRP-conjugated secondary antibody, Santa Cruz Biotechnology, Dallas, TX, United States) was added for blot detection. Signals were detected using a chemiluminescent substrate (GE Healthcare, Little Chalfont, United Kingdom).

### pcDNA3.3-miR146a Cloning

To study the effect of mature *miR146a* on the NF-κB pathway in GBM cell lines, DNA fragments containing primary precursors of *miR146a* were inserted into a pcDNA3.3 mammalian expression vector. The constructed vector pcDNA3.3-mR-146a has been previously designed (Santos et al., 2017). Briefly, the primary precursor of *miR146a* sequence was PCR amplified using the following primer set: 5′ (KpnI)-GCGGTACCGTTTATAACTCATGAGTGCC-3′ and 5′ (XhoI)-ATCTCGAGCTTATACCTTCAGAGCCTG-3′.

### NF-κB-Luciferase Assay

U87MG and T98G cells were transfected with 10 ng of the NF-κB promoter/luciferase reporter plasmids plus 2 ng of the pRL Renilla as control vector (both vectors were gently donated by Dr. Ricardo CT Aguiar from University of Texas Health Center at San Antonio, TX, United States) with Lipofectamine^^®^^ 2000 reagent (Invitrogen, Carlsbad, CA, United States), without FCS and antibiotics. Eight hours after transfection, cells were treated with lipopolysaccharide (LPS; 10 μg/ml) plus 2% FCS, DHMEQ (10 μg/ml) or DPG (U87MG 18 mM; T98G 24 mM) for 40 h. Basal luciferase activity was examined in cells transfected with both NF-κB promoter/luciferase reporter plus pRL Renilla. Luciferase activity was assayed using the dual-luciferase assay kit (Promega, Madison, WI, United States) according to the manufacturer’s instructions. Luminescence was measured with a GloMax^TM^ 96 microplate luminometer (Promega). Three independent transfections were performed for each compound in triplicate and data are shown as the mean ± standard deviation (SD).

Also, U87MG and T98G cells were transfected with 1 μg of the pcDNA3.3-*miR146a* cloning vector, 10 ng of the NF-κB promoter/luciferase reporter plasmid, and 2 ng of the pRL Renilla as control vector with Lipofectamine^^®^^ 2000 reagent (Invitrogen). Eight hours after transfection, cells were treated with DPG (U87MG 18 mM; T98G 24 mM) for 40 h. After, half of the cells were harvested for the *miR146a* and *TRAF6* (Table 1) expression using Trizol^^®^^ and half were used for the luciferase activity as described previously. The expression level of mature *miR146a* was measured by Taqman-based microRNA assay (Applied Biosystems, Foster City, CA, United States) with the use of the ABI Step One system (Applied Biosystems) normalized by U6. Relative quantification of the target was determined by the delta-delta cycle threshold (ΔΔCt) method. The expression level of *TRAF6* was evaluated using SYBR green PCR master mix reagents (Applied Biosystems). Specific primer for this gene (Table 1) and a control without template was included in each plate. The mRNA levels of this gene were normalized to *GAPDH.* For results confirmation, SYBR green PCR was also performed using the *18S* reference gene (Table 1) by the 2^-ΔΔCt^ method.

**Table 1:** Quantitative real-time polymerase chain reaction (PCR) primer sequences.

Gene	Sequence
TRAF6	
Forward primer	5′-CCCAATTCCATGCACATTCAG-3′
Reverse primer	5′-GTTTGAGCGTTATACCCGACT-3′
IRAK2	
Forward primer	5′-TGGCAAATGGTTCCCTACAG-3′
Reverse primer	5′-CATCCACAGCAACGTCAAGA-3′
PARP-1	
Forward primer	5′-GACGTCCCCCAGTGCAGTAAT-3′
Reverse primer	5′-TAGCTGATGGCATGGTGTTC-3′
18S	
Forward primer	5′-CGCGGTTCTATTTTGTTGGT-3′
Reverse primer	5′-CGGTCCAAGAATTTCACCTC 3′

### Quantitative Real-Time Polymerase Chain Reaction (qPCR)

U87MG and T98G cell lines were exposed to 18 and 24 mM DPG for 48 h, respectively. Total RNA from GBM cell lines were isolated using Trizol^^®^^ (Invitrogen) according to the manufacturer’s instructions. The *miR146a*, *miR16*, and U6 cDNAs were synthesized from total RNA according to the TaqMan real-time assays protocol (Applied Biosystems). Relative quantification of the target was determined by the ΔΔCt method. Each sample was examined in triplicate and the raw data were presented as the relative quantity of the target, normalized by U6.

In addition, the quantitative real-time polymerase chain reactions (qPCRs) were performed to evaluate *TRAF6*, *IRAK2*, and *PARP-1* gene expression using SYBR green PCR master mix reagents (Applied Biosystems). Specific primers for each gene (Table 1) and a control without template were included in each plate. Each sample was examined in triplicate and the mRNA levels of each gene were normalized to *GAPDH*. Again, for results confirmation, SYBR green PCR was also performed using the *18S* reference (Table 1) by the 2^-ΔΔCt^ method, for *IRAK2* and *TRAF6* genes.

### Assay to Measure Wound Healing

U87MG and T98G cells (1.0 × 10^6^ cells/well) were seeded in 6-well culture plates and allowed to reach confluence. A 200-μl pipette tip was used to create a wound on the cell monolayer. Then, cells were washed with PBS and cultured with or without exposure to DPG (U87MG 18 mM; T98G 24 mM). Cells were photographed at 0, 24, 48, and 72 h, and ImageJ software (National Institutes of Health, Bethesda, MD, United States) used to measure scratched areas. The same wound areas were observed and photographed under an inverted microscope (Axio Vert. A1 ZEISS). The distance of the scratch closure was examined at 0, 24, 48, and 72 h.

### Neuro-Sphere Formation Assay

To culture cell line spheres, 10,000 cells/ml U87MG and T98G were seeded in 2% poly(2-hydroxyethyl methacrylate) (poly-HEMA, Kasvi, São José dos Pinhais, Paraná, Brazil)-coated cell culture flasks to prevent cell adhesion. Neuro-spheres were formed in serum-free medium. Serum-free spheres were cultured in DMEM/F12 supplemented with N2 supplement (StemCell, Vancouver, Canada). Epidermal growth factor (EFG) and basic fibroblast growth factor (FGFb) (20 ng/ml each) (Peprotech, Ribeirão Preto, São Paulo, Brazil) were added to the medium before culturing. Cell culture medium was changed every 3 days. Neuro-spheres were cultured at least 6 days in serum-free medium. All cells were cultured at 37°C in a humidified atmosphere of 5% CO_2_. For subsequent DPG (U87MG: 18 mM; T98G: 24 mM) treatment, 75-μm neuro-spheres were cultured for 24, 48, and 72 h. Cells without DPG were cultured as controls. Cells were observed and photographed under an inverted microscope (Axio Vert. A1 ZEISS).

### Statistical Analysis

A two-tailed *t*-test was performed for all two sets of numerical data (treated and non-treated cells) and *P* ≤ 0.05 was considered statistically significant. Results are expressed as mean ± SD from experiments repeated at least three times. For gene expression analysis, the data set was probed for normality using Shapiro–Wilk’s test. Because the data sets did assume normal distribution, *t*-test was performed for the comparison of groups. All tests were done using the SPSS 21.0 software (SPSS Incorporation, Chicago, IL, United States).

## Results

### Inhibitory Effect of DPG on Cell Viability

**FIGURE 1:**
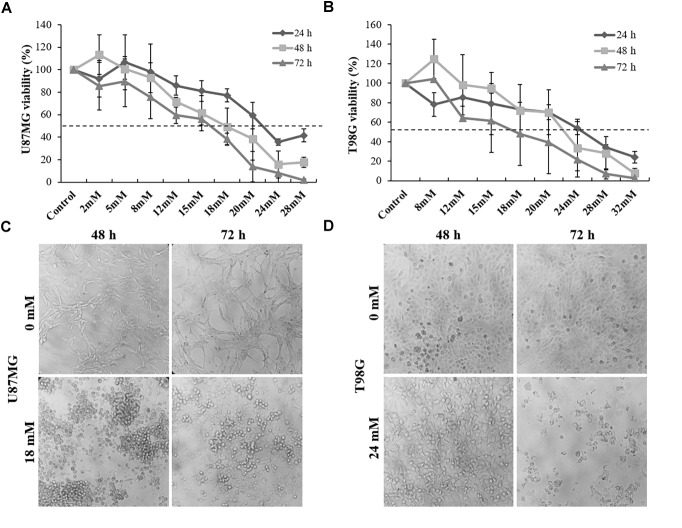
Dipotassium glycyrrhizinate (DPG) reduces cell viability and change morphology in glioblastoma cell lines. **(A)** DPG inhibits cell viability of U87MG cells treated with 12, 15, 18, 20, 24, and 28 mM DPG for 24, 48, and 72 h by MTT assay and the IC_50_ was determined (18 mM for 48 and 72 h). **(B)** DPG inhibits cell viability of T98G cells treated with 20, 24, 28, and 32 mM DPG for 24, 48, and 72 h by MTT assay and the IC_50_ was determined (24 mM for 48 and 72 h). **(C)** U87MG cell and nuclear morphological change were observed 48 and 72 h after DPG exposure. **(D)** T98G cell and nuclear morphological change were observed especially after 72 h of DPG exposure.

The present study examined the effectiveness of DPG as an anti-tumor in U87MG and T98G GBM cell lines. Thus, no significant reduction in cell viability was detected when published-based concentrations of DPG were added to GBM cell lines (100 μM, 300 μM, 500 μM, 700 μM, 1,200 μM, 1,600 μM, and 2 mM) (Supplementary Figures S1A,B). In addition, no significant reduction in cell viability was detected when low concentrations of DPG were added to U87MG (2, 5, and 8 mM) and T98G (8, 12, and 15 mM) cells for 24, 48, and 72 h. However, an inverse relationship was observed between GBM cell viability and high concentrations of DPG (12, 18, 20, 24, 28, and 32 mM) especially following 48- and 72-h treatments (Figure 1A,B). The IC_50_ concentrations of DPG on U87MG cells at 24, 48, and 72 h were 28 mM (*P* = 2E^-08^), 18 mM (*P* = 0.0001), and 18 mM (*P* = 6E^-11^), respectively (Figure 1A). The IC_50_ concentrations of DPG on T98G cells at 24, 48, and 72 h were 24 mM (*P* = 0.01), 24 mM (*P* = 0.08), and 18 mM (*P* = 0.01), respectively (Figure 1B).

For all further assays, the adopted IC_50_ concentrations for U87MG and T98G cells were 18 and 24 mM for 48 h, respectively. Interestingly, nuclear and cell morphology changes, such as condensation and fragmentation of nuclei and cells, were observed in both cell lines after DPG treatment when compared to the control (Figure 1C,D).

### Synergistic Effect of DPG With TMZ on the Viability of GBM Cell Lines

U87MG cells showed reduction in cell viability after 168 h with all lower TMZ concentrations, but not significantly (*P* > 0.05). Specifically, 75 μM presented highest reduction in cell viability (*P* = 0.56) (Supplementary Figure S1C). When higher TMZ concentrations were used, U87MG cells exhibited reduction in cell viability only after 96 h of TMZ treatment (500 μM) and T98G cells presented cell viability reduction after 96 h (2,000 μM) (*P* = 0.01 and *P* = 0.001, respectively) (Supplementary Figures S1D,E).

When low TMZ concentrations with IC_50_ DPG were tested, we observed significant U87MG and T98G cell viability reduction for all doses (25–200 μM) and incubation times (6, 12, 18, and 24 h) tested, starting at 25 μM for 6 h of treatment (*P* = 0.009 and 2.2E^-05^, respectively) (Supplementary Figures S2A,B).

Once the apoptosis-inducing effect of DPG starts to diminish after 72 h of treatment, and cells show proliferative characteristics again, higher TMZ concentrations (250–2,000 μM) were also tested in combination with IC_50_ DPG with higher incubation times (24, 48, 72, and 96 h) for both cell lines. Interestingly, TMZ in combination with IC_50_ DPG was a stronger inducer of U87MG and T98G cell growth arrest than TMZ alone for all tested doses, starting from the concentration of 250 μM for 24 h of treatment (*P* = 0.003 and *P* = 1.0E^-07^, respectively). We found that combined with DPG, TMG significantly reduced cell viability in a dose- and time-dependent manner in comparison with the control (Supplementary Figures S2C,D), revealing a synergistic effect when cells were simultaneously treated at all concentrations of TMZ.

### DPG Induces DNA Fragmentation

**FIGURE 2:**
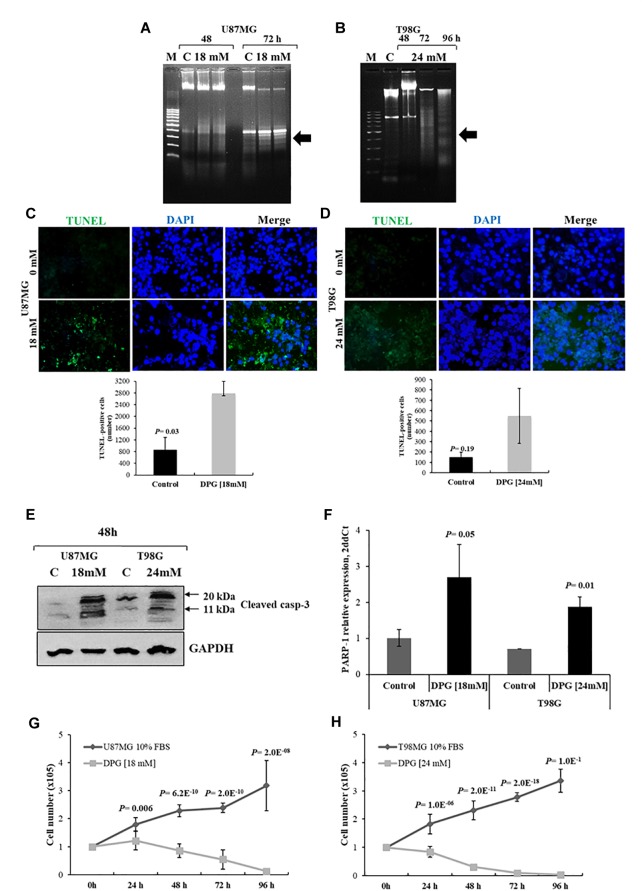
Dipotassium glycyrrhizinate (DPG) increases apoptosis and inhibits proliferation in glioblastoma cell lines. U87MG **(A)** and T98G **(B)** cells were incubated with 18 mM and 24 mM of DPG, respectively, for 48 and 72 h. T98G was also treated using 24 mM DPG for 96 h. The genomic DNA was isolated and analyzed on 1.5% agarose gel with ethidium bromide staining. M: DNA marker 100 base pairs; C: untreated control cells. Results are representative of one of three similar experiments performed. U87MG **(C)** and T98G **(D)** cells were treated with DPG (18 mM for 72 h and 24 mM for 96 h of DPG, respectively). After DPG incubation TUNEL assay was done using *in situ* cell death detection kit as per the manufacturer’s protocol and the quantitative estimation of TUNEL cells after DPG exposure was measured using ImageJ. Each experiment was repeated three times. The results show a significant increase in the number of apoptotic U87MG cells (*P* = 0.03) after DPG treatments as compared to the untreated control cells. T98G (*P* = 0.19) cell also presents an increase in the apoptotic cell number. **(E)** Cleaved caspase-3 was measured by Western blotting analysis after treatment with 18 mM (U87MG) and 24 mM (T98G). The induction of the cleaved form of caspase-3 (11 kDa) protein increased 48 h after treatment. DPG increased cleaved caspase-3, indicating that apoptosis required caspase activation. **(F)** Also, DPG significantly increases *PARP-1* expression in U87MG (*P* = 0.05) and T98G (*P* = 0.01) cells when compared with untreated cells. DPG inhibits the proliferating rate of U87MG **(G)** and T98G **(H)** cell lines in a time-dependent trend, in comparison with untreated control cells. Data represent means and standard deviations of a representative experiment performed in triplicate. Statistics were performed in a two-tailed *t*-test with *P* < 0.01.

We examined DNA fragmentation to confirm apoptosis in cells treated with DPG. U87MG cells were treated with 18 mM of DPG for 48 and 72 h. T98G cells were treated with 24 mM of DPG for 48, 72, and 96 h. DNA was isolated and analyzed by agarose gel electrophoresis. As shown in Figure 2A, DPG treatment induced DNA degradation in U87MG cells after 72 h of DPG treatment in comparison with control. In addition, DPG induced DNA degradation in T98G cells especially after 96 h of treatment in comparison with control (Figure 2B). Those results suggested that the cells treated with DPG may be undergoing apoptosis.

### DPG Induces Apoptosis and Inhibits Proliferation of GBM Cell Lines

To confirm whether DPG induced DNA degradation in GBM cell lines, TUNEL assay was used to identify and quantify apoptotic cells. U87MG cells were treated with 18 mM of DPG for 72 h. T98G cells were treated with 24 mM of DPG for 96 h. Disrupted DNA was stained by green fluorescence and cell nuclei were visualized by DAPI counterstaining. As shown in Figure 2C, the U87MG cell line presented an increased number of TUNEL-positive cells compared to the control (*P* = 0.03). T98G-treated cells showed no significant difference when compared to the control cells (*P* = 0.19), although some TUNEL-positive cells were observed only in the DPG-treated cells (Figure 2D).

Also, cleaved caspase-3 was verified by Western blot in both cell lines after DPG addition (U87MG: 18 mM; T98G: 24 mM), indicating that the cells treated with DPG suffered apoptosis, in accordance with DNA fragmentation and TUNEL results (Figure 2E). Since PARP-1 is one of several known cellular substrates of caspase-3 and cleavage of PARP-1 by caspase-3 is considered to be a hallmark of apoptosis, we also performed *PARP-1* gene expression by qPCR after DPG treatment (U87MG: 18 mM; T98G: 24 mM). *PARP-1* expression level was significantly higher in U87MG and T98G cells exposed to DPG when compared with untreated cells [2.71 vs. 1.01 arbitrary units (AUs), *P* = 0.05; 1.88 vs. 0.71 AUs, *P* = 0.01, respectively] (Figure 2F).

As exhibited in Figure 2G,H, counting assay indicated a significant inhibition of proliferating rate in U87MG and T98G cells treated with DPG in a time-dependent trend (*P* < 0.05), in comparison with untreated control cells.

### DPG Regulates NF-κB Activity in GBM Cell Lines

**FIGURE 3:**
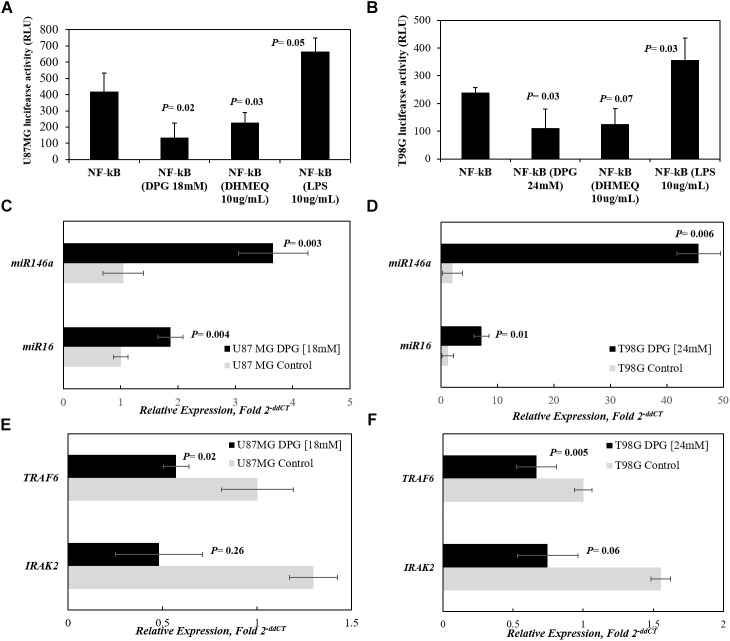
DPG regulates NF-κB activity in glioblastoma cell lines by *miR16*- and *miR146a*-mediating *IRAK2* and *TRAF6* down-expression. U87MG **(A)** and T98G **(B)** cell lines were transfected with the NF-κB-luciferase reporter and incubated with dipotassium glycyrrhizinate (DPG) for 40 h. Equal amounts of cell extract were assayed for dual-luciferase activity. The reporter activity was observed as down-regulated by DPG when comparing with untreated transiently transfected cells. Transiently transfected cells were also treated with dehydroxymethylepoxyquinomicin (DHMEQ) or lipopolysaccharide (LPS). As expected, DHMEQ and LPS down-regulates and induces NF-κB reporter activity, respectively, in both cell lines. DPG significantly increases *miR16* and *miR146a* expression in U87MG (*P* = 0.004 and *P* = 0.003, respectively) **(C)** and T98G (*P* = 0.01 and *P* = 0.006, respectively) cell lines. **(D)** Instead, DPG decreases *IRAK2* and *TRAF6* mRNA levels in U87MG (*P* = 0.26 and *P* = 0.02, respectively) and **(E)** T98G (*P* = 0.01 and *P* = 0.005, respectively) cells. **(F)** Data represent means and standard deviations of a representative experiment performed in triplicate. Statistics were performed in a two-tailed *t*-test with *P* ≤ 0.05.

We further investigated the effect of DPG on NF-κB activity in GBM cancer cells. U87MG and T98G cells were transiently transfected with NF-κB-Luc reporter plasmid, and the reporter activity was found to be down-regulated by DPG in comparison with untreated transfected cells (*P* = 0.02 and *P* = 0.03, respectively) (Figure 3A,B). Also, transiently NF-κB-Luc-transfected cells were treated with LPS or DHMEQ. As expected, the LPS-induced cells significantly increases in NF-κB reporter activity in U87MG (*P* = 0.05) and in T98G (*P* = 0.003). DHMEQ treatment, a compound that has shown to inhibit NF-κB DNA binding and transcriptional activity in GBM cells, significantly down-regulated NF-κB in U87MG (*P* = 0.03). DHMEQ inhibited NF-κB transcriptional activity in T98G (*P* = 0.07). Interestingly, in a similar way, DPG inhibited NF-κB in comparison with untreated cells (*P* = 0.02 and *P* = 0.03, in U87MG and T98G, respectively) (Figure 3A,B).

### DPG Up-Regulates miR-16 and miR146a Down-Regulates Its Target Genes in GBM Cells

To identify microRNAs (miRs) involved with the NF-κB signaling pathway, we generated a list with previously published miRs as transcriptional targets of the NF-κB signaling pathway. Next, the miRs included in the list were analyzed in the databases TargetScan^1^ and mirDB^2^ to check for predicted gene targets belonging to the NF-κB. *MiR16* and *miR146a* were selected due to two criteria: >95% probability to bind *IRAK2* and *TRAF6* genes, respectively, and their involvement with GBM described in previous studies. Moreover, it is important to highlight that *IRAK2* and *TRAF6* are validated *miR146a* targets (Taganov et al., 2006).

Thus, the present study determined whether the effect of cytotoxicity of DPG in GBM cells is mediated through up-regulation of *miR16* and *miR146a* by quantitative real-time polymerase chain reaction (qPCR). The mean *miR16* and *miR146a* expression levels were significantly higher in DPG exposure U87MG cells when compared with untreated cells (1.87 vs. 1.01 AUs, *P* = 0.004; 3.66 vs. 1.05 AUs, *P* = 0.003, respectively) (Figure 3C). Also, the mean *miR16* and *miR146a* expression levels were higher in DPG-treated T98G cells when compared with untreated cells (7.18 vs. 1.23 AUs, *P* = 0.01; 45.60 vs. 2.03 AUs, *P* = 0.0006, respectively) (Figure 3D). The results showed that DPG significantly increased *miR16* and *miR146a* expression in GBM cell lines.

In this study, we also found that treatment with DPG in U87MG and T98G cells significantly decreased *TRAF6* mRNA level (0.57 vs. 1.00 AUs, *P* = 0.02; 0.67 vs. 1.00 AUs, *P* = 0.005, respectively) (Figure 3E,F). Otherwise, *IRAK2* mRNA level presented non-significant reduction in U87MG and T98G DPG exposure cells compared to control cells (0.48 vs. 1.30 AUs, *P* = 0.26; 0.75 vs. 1.55 AUs, *P* = 0.06, respectively) (Figure 3E,F). These data indicate that DPG can inhibit cell proliferation and invasion by up-regulating *miR16* and *miR146a* and down-regulating its target genes.

As expected, qPCR results using *18S* reference were similar and the mean *TRAF6* and *IRAK2* expression levels were significantly decreased in U87MG cells treated with DPG compared to untreated cells (0.30 vs. 1.06 AUs, *P* = 0.02; 0.44 vs. 1.01 AUs, *P* = 0.003, respectively) (Supplementary Figure S3A). Also, the mean *TRAF6* and *IRAK2* expression levels were lower in T98G-treated cells when compared with untreated cells (0.28 vs. 1.00 AUs, *P* = 0.03; 0.20 vs. 1.01 AUs, *P* = 0.008, respectively) (Supplementary Figure S3B).

Since the difference in expression levels after DPG treatment was significantly higher for *TRAF6*-mediating *miR146a* stimulation, we tested next whether *miR146a* overexpressing could influence the inhibitory effect of DPG on suppressing the NF-κB pathway.

### MiR-146a Enhances the Effect of DPG by Suppressing the NF-κB Pathway

**FIGURE 4:**
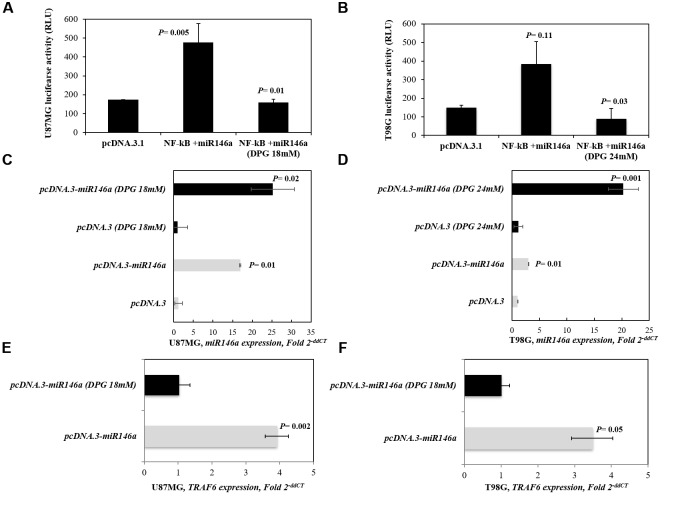
Inhibitory effect of dipotassium glycyrrhizinate (DPG) in the NF-κB signaling pathway. pcDNA3.3-empty vector and pcDNA3.3-*miR146a* cloning vector were transiently transfected into U87MG and T98G cell lines and treated with DPG (U87MG: 18 mM; T98G: 24 mM). **(A)** The promoter activity of NF-κB in U87MG-pcDNA3.3-*miR146a* (*P* = 0.005) presented significantly higher compared to pcDNA3.3-empty vector cells. In contrast, the NF-κB activity presented significantly reduced when U87MG-pcDNA3.3-*miR146a* (*P* = 0.01) cells were treated with DPG. **(B)** The promoter activity of NF-κB in T98G-pcDNA3.3-*miR146a* (*P* = 0.11) is higher compared to T98G-pcDNA3.3-empty vector cells. Instead, the NF-κB promoter activity presented significantly reduced when T98G-pcDNA3.3-*miR146a* (*P* = 0.03) cells were treated with DPG. *MiR146a* expression was evaluated by qPCR. *MiR146a* is higher in either **(C)** U87MG- and **(D)** T98G-pcDNA3.3-*miR146a* untreated cells (*P* = 0.01 and *P* = 0.01, respectively) and U87MG- and T98G-pcDNA3.3-*miR146a* treated with DPG (*P* = 0.02 and *P* = 0.001, respectively). DPG decreases *TRAF6* mRNA level in either **(E)** U87MG- or **(F)** T98G-pcDNA3.3-*miR146a* (*P* = 0.002 and *P* = 0.05, respectively) cells. Three independent transfections were performed in triplicate, and data are shown as the mean ± standard deviation. Statistics were performed in a two-tailed *t*-test with *P* ≤ 0.05.

We investigated the effect of DPG on NF-κB activity in U87MG- and T98G-pcDNA3.3-*miR146a*-transfected cell lines. As expected, the promoter activity of NF-κB in U87MG-pcDNA3.3-*miR146a* (*P* = 0.005) presented significantly higher compared to U87MG-pcDNA3.3-empty vector in unexposed cells. Also, the activity of NF-κB was higher in T98G-pcDNA3.3-*miR146a* cells (*P* = 0.11) than in T98G-pcDNA3.3-empty vector untreated cells. Interestingly, when U87MG- and T98G-pcDNA3.3-*miR146a*-transfected cell lines were treated with DPG, we observed that the promoter activity of NF-κB significantly decreased when compared with pcDNA3.3-empty vector cell lines (*P* = 0.01 and *P* = 0.03, respectively) (Figure 4A,B).

The pcDNA3.3-empty vector and pcDNA3.3-*miR146a*-transfected cells were treated with DPG (U87MG: 18 mM; T98G: 24 mM). Untreated transfected cells were used as controls. The *miR146a* expression was analyzed by qPCR. Thus, *miR146a* expression presented similar in U87MG and T98G cells transfected with pcDNA3.3-empty vector treated with DPG when compared with untreated cells (1.23 vs. 1.01 AUs, *P* = 0.79; 1.15 vs. 1.00 AUs, *P* = 0.81, respectively). Moreover, *miR146a* expression level presented higher in U87MG-pcDNA3.3-*miR146a* (Figure 4C) and T98G-pcDNA3.3-*miR146a* cells (Figure 4D) upon exposure to DPG compared to untreated GBM-pcDNA3.3-*miR146a*-transfected cells (25.30 vs. 17.00 AUs, *P* = 0.19; 20.27 vs. 3.00 AUs, *P* = 0.01, respectively).

Also, U87MG- and T98G-pcDNA3.3-*miR146a* cells treated with DPG significantly resulted in decreased *TRAF6* expression level (1.02 vs. 3.95 AUs, *P* = 0.002; 1.12 vs. 3.64 AUs, *P* = 0.05, respectively) (Figure 4E,F). The results using *18S* as reference were similar in U87MG-pcDNA3.3-*miR146a*- and T98G-pcDNA3.3-*miR146a*-treated and untreated cells (0.53 vs. 1.00 AUs, *P* = 0.03; 0.27 vs. 1.01 AUs, *P* = 0.04, respectively) (Supplementary Figures S3C,D).

### DPG Inhibits the Migration Ability of GBM Cells

**FIGURE 5:**
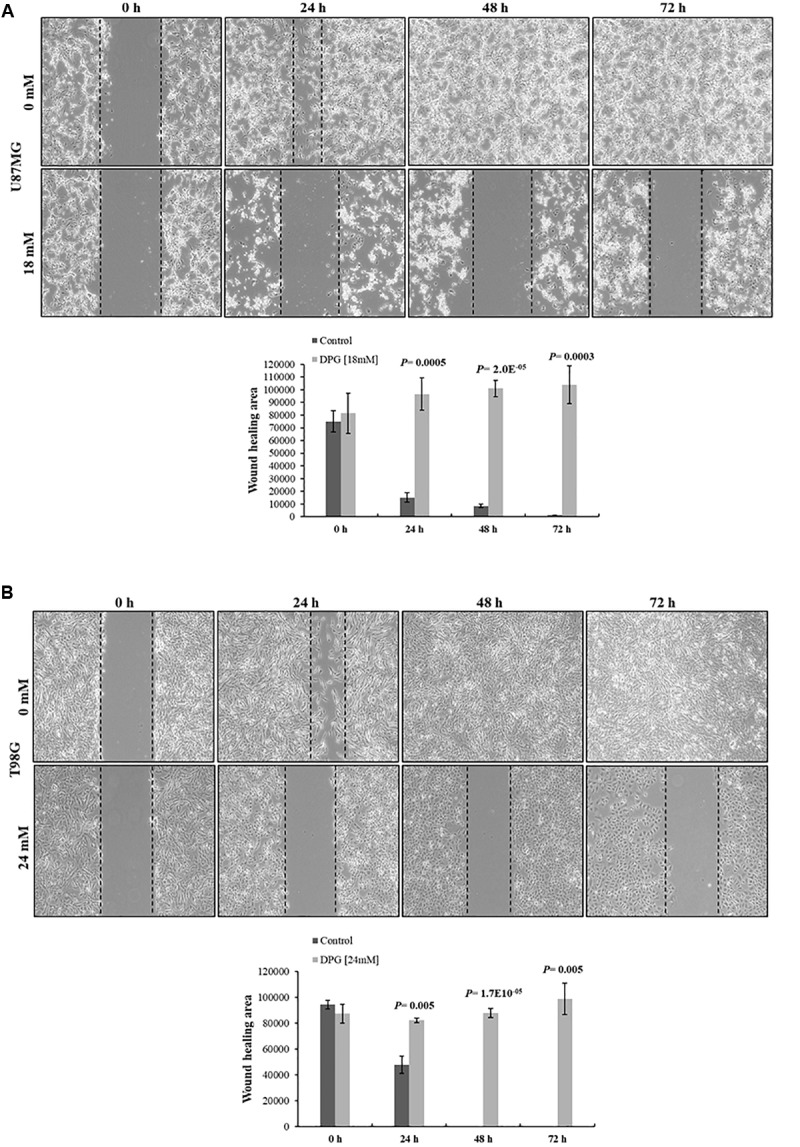
Dipotassium glycyrrhizinate (DPG) decreases wound closure and inhibits cell migration in glioblastoma cell lines. **(A)** U87MG cell line treated with DPG fills the wound area (the area between the two dotted lines) more slowly than those untreated at 24, 48, and 72 h. **(B)** T98G cell line treated with DPG fills the wound area (the area between the two dotted lines) more slowly than those untreated at 24, 48, and 72 h. The wound-healing assay was expressed as relative wound width (24, 48, or 72 h average wound width divided by 0 h wound represented by the graphics). Graphics also shows average and standard deviation of three independent experiments. Statistics were performed in a two-tailed *t*-test with *P* ≤ 0.05.

To investigate the effect of DPG on the migration ability of GBM cells, U87MG and T98G cells were treated with DPG for 24, 48, and 72 h and scratch wound healing motility assay was performed simultaneously. The results showed that U87MG and T98G cell lines treated with 18 mM and 24 mM of DPG for 24, 48, and 72 h migrated significantly slower than medium DPG-free control cells (Figure 5A,B), suggesting that DPG could significantly decrease migratory ability through overexpression of *miR146a* and *miR16*, while the NF-κB pathway is reduced in U87MG and T98G cells.

### DPG Reduces Neuro-Sphere Formation

**FIGURE 6:**
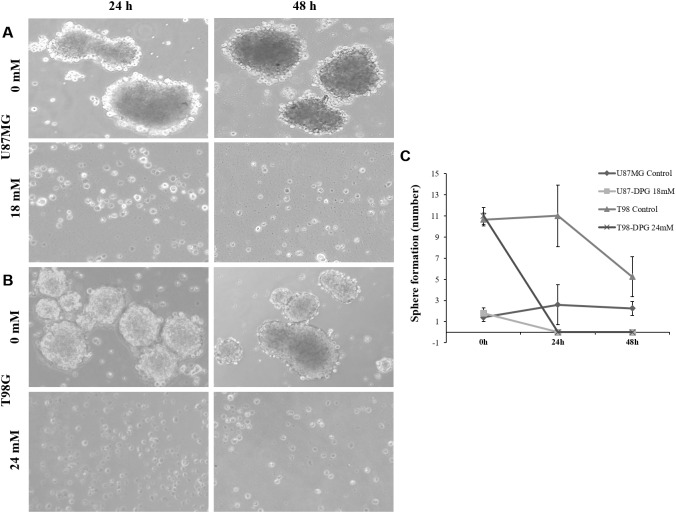
Glioblastoma stem cells (GSCs) are sensitized to dipotassium glycyrrhizinate (DPG). Sphere-forming ability of GSCs in U87MG **(A)** and T98G **(B)** cell lines after 24 and 48 h after DPG treatment. Non-treated cell lines were used as controls. Cells were plated in 2% poly (2-hydroxyethyl methacrylate)-coated cell culture flasks in serum-free medium. **(C)** The graphic shows the average and standard deviation of three independent experiments. Statistics were performed in a two-tailed *t*-test with *P* ≤ 0.05. Differences between treated and non-treated cells were significantly observed in U87MG (*P* = 0.04) and T98G (*P* = 1E^-04^) cell lines.

In the neuro-sphere formation assay, cells were plated with and without DPG, and the number of neuro-spheres formed was counted from days 4 to 6 after plating. In the presence of DPG, there were no neuro-spheres in U87MG and T98G cell lines after 24 h treatment (*P* = 0.04 and *P* = 1E^-04^, respectively) (Figure 6A–C), indicating that DPG reduces the relative number of brainstem cells capable of forming neuro-spheres.

## Discussion

Deregulation of the NF-κB pathway promotes GBM tumor growth and progression through the transcriptional activation of genes associated with suppression of apoptosis, metastasis, and resistance to cytotoxic agents (Gilmore and Garbati, 2011). Recently, a variety of natural and synthetic molecules that inhibit activation of NF-κB have been identified, which have shown growth inhibitory effects in GBM (Aggarwal, 2004; Robe et al., 2004; Dhandapani et al., 2007; Kesanakurti et al., 2009; Aguilar-Morante et al., 2010; Fujiwara et al., 2011; Zanotto-Filho et al., 2011). Licorice root’s extract has been used for centuries as treatment for ulcers, inflammation, viral infections, arthritis, as well as cancer (Wang and Nixon, 2001; Shim et al., 2012; Vitali et al., 2013). There are numerous reports demonstrating that G, an active compound in licorice root, inhibited cancer cell lines grown *in vitro* (Abe et al., 1987; Chung et al., 2000; Kobayashi et al., 2002; Cassileth and Deng, 2004; Cragg and Newman, 2005; Hibasami et al., 2005, 2006; Ikeda et al., 2006; Menegazzi et al., 2008; Khan et al., 2013; Huang et al., 2014). GA, another metabolite of G, was found to reduce the prolife- ration rate of prostate cancer cells (Sivasakthivel et al., 2008).

In the present study, we analyzed the effects of NF-κB signaling pathway inhibition by DPG, a dipotassium salt of GA, known for its anti-inflammatory properties (Cosmetic Ingredient Review Expert Panel, 2007; Shim et al., 2012; Vitali et al., 2013; Li et al., 2014), on the survival, chemoresistance, neuro-sphere formation, and migration of GBM cell lines, U87MG, and T98G. To the best of our knowledge, this is the first report to demonstrate the potential role of DPG as anti-tumor action on GBM.

We also assessed the apoptotic effects of DPG in GBM cells and investigated the possible molecular mechanisms. We showed that DPG significantly inhibits U87MG and T98G cell growth in a dose- and time-dependent manner. Apoptosis rate is considered an indicator of anticancer activity. Activation of caspases is a central process of the two major apoptosis pathways, where activated caspases provide a link between cell signaling and apoptotic execution (Mantovani et al., 2008). Total caspase-3 is activated under apoptotic conditions that interact with pro-apoptotic caspases-8 and -9 and also cleaves and activates caspases-6 and -7 (Wang et al., 2003). These findings indicated that DPG inhibits cell growth through induction of apoptosis.

Poor clinical response and patient outcome in GBM are mostly due to chemo- and radioresistance (Iacob and Dinca, 2009). The involvement of NF-κB in anticancer drug resistance has been described in various *in vitro* and *in vivo* models (Wang et al., 1999); thereby, its inhibition could potentially lead to the reversal of chemoresistance. In the present study, we suggested significantly synergistic effects when cells were simultaneously treated with TMZ and DPG in comparison with GBM cells only exposed to TMZ. Thus, when cell lines were exposed to clinically relevant TMZ concentrations (25–200 μM), an interesting synergistic effect was observed, where DPG was able to sensitize the GBM cells to TMZ, with TMZ becoming effective at concentrations or incubation times lower than those observed when used alone. When higher TMZ concentrations (250–2,000 μM) were tested, half-dose of TMZ associated with DPG reduces cell viability three times faster than TMZ alone, revealing synergistic effects.

Dipotassium glycyrrhizinate inhibited cell growth and induced apoptosis of GBM cells *in vitro* by overexpressing *miR146a* and *miR16* through the NF-κB signaling pathway. The expression level of *miR16* positively correlated with GBM stem cell differentiation, but negatively with the abilities of migration, motility, invasion, and colony formation in GBM cells (Wang et al., 2014; Tian et al., 2017). Also, previously, *miR16* overexpression reduced the promoter activity of NF-κB in U87MG and U251 cell lines (Wang et al., 2014; Tian et al., 2017). Our results also showed that *miR16* overexpression after DPG inhibited *IRAK2*, indicating NF-κB suppressing by DPG. *MiR146a* is known as a negative regulator of NF-κB signaling that affects tumor growth and survival of GBM cell lines (Wu et al., 2015). Here, we showed that overexpression of *miR146a* after DPG stimulation influenced *TRAF6* down-expression. Therefore, we believed that *miR146a* may positively inhibit NF-κB through suppressing *TRAF6* after DPG action. In this context, *IRAK2*- and *TRAF6*-mediating *miR16* and *miR146a*, respectively, might be a potential therapeutic target of DPG.

Our results were most significant in U87MG cell lines. The U87MG and T98G are well-known TMZ-sensitive and -resistant GBM cell lines, respectively (Sang, 2016; Wu et al., 2015).

Glioblastoma is characterized by having a cancer stem cell subpopulation essential for tumor formation, survival, and recurrence (Reya et al., 2001; Ledur et al., 2012). Indeed, cancer stem cells are also responsible for chemoresistance (Zong et al., 2015). Therefore, identifying agents that target brain cancer stem cells is a promising strategy in the development of chemotherapeutics to treat GBM.

Several inhibitors of neuro-sphere proliferation were evaluated in a broad chemical screen, and several compounds presented inhibitory effects on the proliferation of cultures enriched for brain cancer stem cells. These chemicals induced different neuro-sphere phenotypes, altering sphere number, size, and adhesion properties (Diamandis et al., 2007). Previously, studies have shown that TMZ, at a sub-toxic concentration, reduced the formation of spheres by 50%, similar to the reduction found in primary GBM tumors cultivated with defined factors (Diamandis et al., 2007; Beier et al., 2008). Our results indicated a formation of neuro-sphere reduction of 100% after 24 h DPG exposure, indicating that DPG should be explored as an alternative treatment since affecting tumor malignity.

Taken all together, we conclude that DPG is able to induce nuclear and cell morphology changes, leading the cells to undergo apoptosis due to caspase-3 activation and DNA fragmentation, which may contribute to their decreased viability. DPG can also inhibit cell proliferation and invasion by up-regulating *miR16* and *miR146a*, which leads to the down-regulation of their target genes, as the NF-κB pathway, significantly decreasing migratory ability. Further follow-up *in vitro* analyses, with other GBM cell lines, and *in vivo* studies may contribute to clarify the role of DPG in GBM.

## Author Contributions

MMO contributed to the conception and design. GAB and JSS conducted the acquisition of data. GAB, JSS, and MMO performed analyses and interpretation of data. TR performed analyses and interpretation of TUNEL assay. MMO drafted the manuscript and performed study supervision. JVZ, KU, TR, MLR, and MMO provided important reagents. All authors were involved in the revision of the manuscript and approved the final version.

## Conflict of Interest Statement

The authors declare that the research was conducted in the absence of any commercial or financial relationships that could be construed as a potential conflict of interest.

## Footnotes

1 www.targetscan.org

2 http://mirdb.org/miRDB/

## Supplementary Material

FIGURE S1 High temozolomide (TMZ) concentrations significantly reduce glioblastoma cell line viability. DPG does not inhibit cell viability of U87MG **(A)** and T98G **(B)** cells treated with 100, 300, 500, 700, 1,200, 1,600, and 2,000 μM DPG for 24, 48, and 72 h by MTT. All experiments were performed in triplicate and were repeated at least twice. The graphic shows the standard deviation of three independent experiments. Statistics were performed in a two-tailed *t*-test with *P* ≤ 0.05. **(C)** TMZ inhibits U87MG cell proliferation after 168 h of treatment (75 μM). **(D)** TMZ inhibits U87MG cell proliferation after 72 h of treatment (500 μM). **(E)** TMZ inhibits T98G cell proliferation after 72 h of treatment (1,750 μM). All experiments were performed in triplicate and were repeated at least twice. Graphics are representative of one of three independent experiments.

FIGURE S2 Temozolomide (TMZ) in combination with dipotassium glycyrrhizinate (DPG) induces significant dose- and time-dependent reductions of cell viability. Low TMZ concentrations with IC_50_ DPG was able to induce U87MG **(A)** and T98G **(B)** cell viability reduction in incubation times (6, 12, 18, and 24 h) lower than those observed when used alone in a dose- and time-dependent manner. High TMZ concentrations with IC_50_ DPG was able to induce U87MG **(C)** and T98G **(D)** cell viability reduction in incubation times (24, 48, 72, and 96 h) lower than those observed when used alone in a dose- and time-dependent manner. The graphic shows the standard deviation of three independent experiments. Statistics were performed in a two-tailed *t*-test with *P* ≤ 0.05. All experiments were performed in triplicate and were repeated at least twice. Graphics are representative of one of three independent experiments.

FIGURE S3 Dipotassium glycyrrhizinate (DPG) down-regulates *IRAK2* and *TRAF6*. **(A)** DPG decreases *IRAK2* and *TRAF6* mRNA levels in U87MG (*P* = 0.02 and *P* = 0.003, respectively) and **(B)** T98G (*P* = 0.03 and *P* = 0.008, respectively) compared to untreated cell lines using 18S reference. DPG decreases IRAK2 and TRAF6 mRNA levels in **(C)** U87MG-pcDNA3.3-miR146a and **(D)** T98G-pcDNA3.3-miR146a (*P* = 0.03 and *P* = 0.04, respectively) compared to untreated pcDNA3.3-miR146a cells using 18S reference. Data represent means and standard deviations of a representative experiment performed in triplicate. Statistics were performed in a two-tailed *t*-test with *P* ≤ 0.05.
