# Supplementary figures and images for: Growth Inhibitory Effects of Dipotassium Glycyrrhizinate in Glioblastoma Cell Lines by Targeting MicroRNAs Through the NF-κB Signaling Pathway
Source: Front Cell Neurosci. 2019 May 28;13:216. doi: 10.3389/fncel.2019.00216 (PMC6546822; doi:10.3389/fncel.2019.00216)

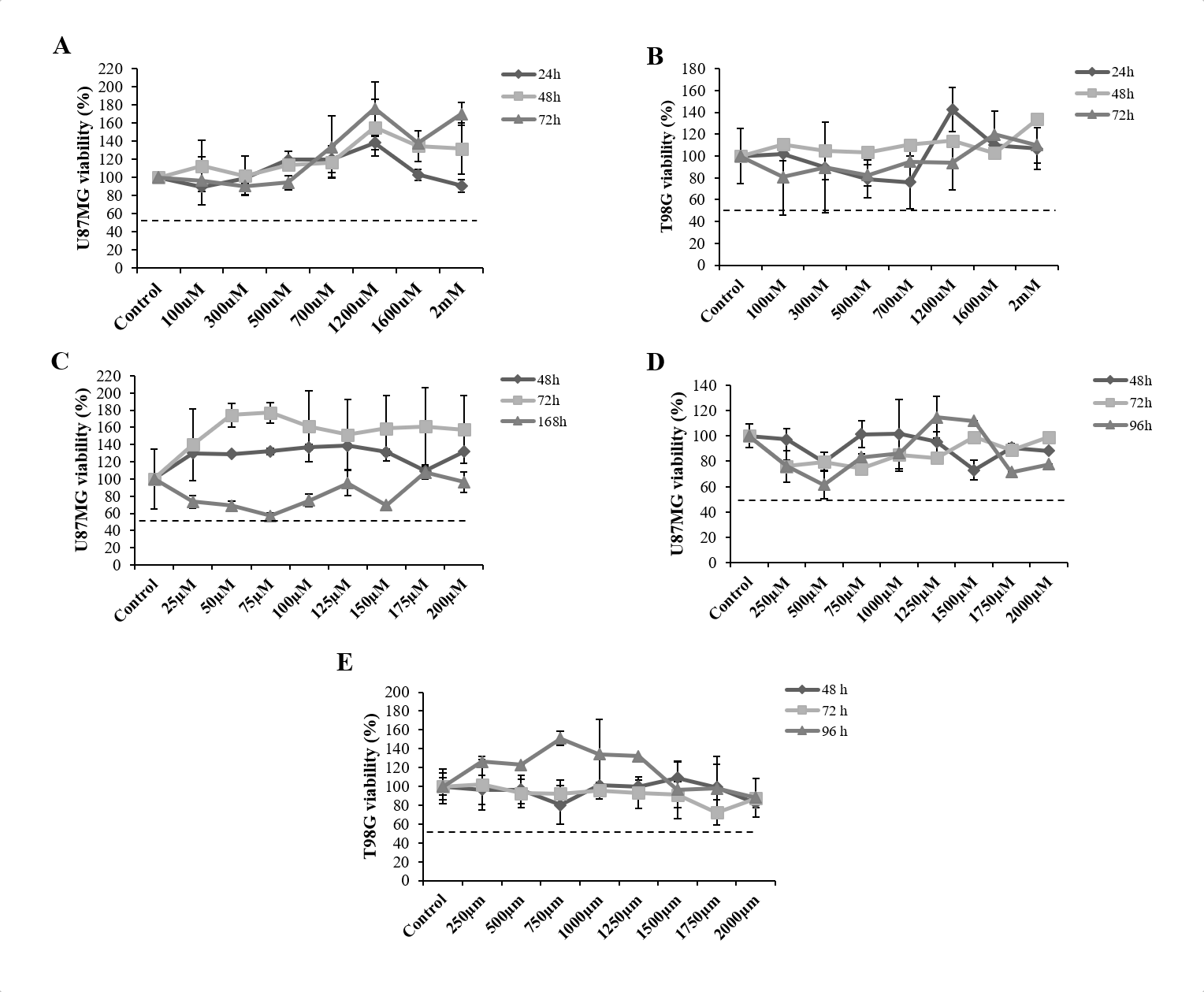

Supplement: FIGURE S1 — High temozolomide (TMZ) concentrations significantly reduce glioblastoma cell line viability. DPG does not inhibit cell viability of U87MG (A) and T98G (B) cells treated with 100, 300, 500, 700, 1,200, 1,600, and 2,000 μM DPG for 24, 48, and 72 h by MTT. All experiments were performed in triplicate and were repeated at least twice. The graphic shows the standard deviation of three independent experiments. Statistics were performed in a two-tailed t-test with P ≤ 0.05. (C) TMZ inhibits U87MG cell proliferation after 168 h of treatment (75 μM). (D) TMZ inhibits U87MG cell proliferation after 72 h of treatment (500 μM). (E) TMZ inhibits T98G cell proliferation after 72 h of treatment (1,750 μM). All experiments were performed in triplicate and were repeated at least twice. Graphics are representative of one of three independent experiments. [file Image_1.TIF]

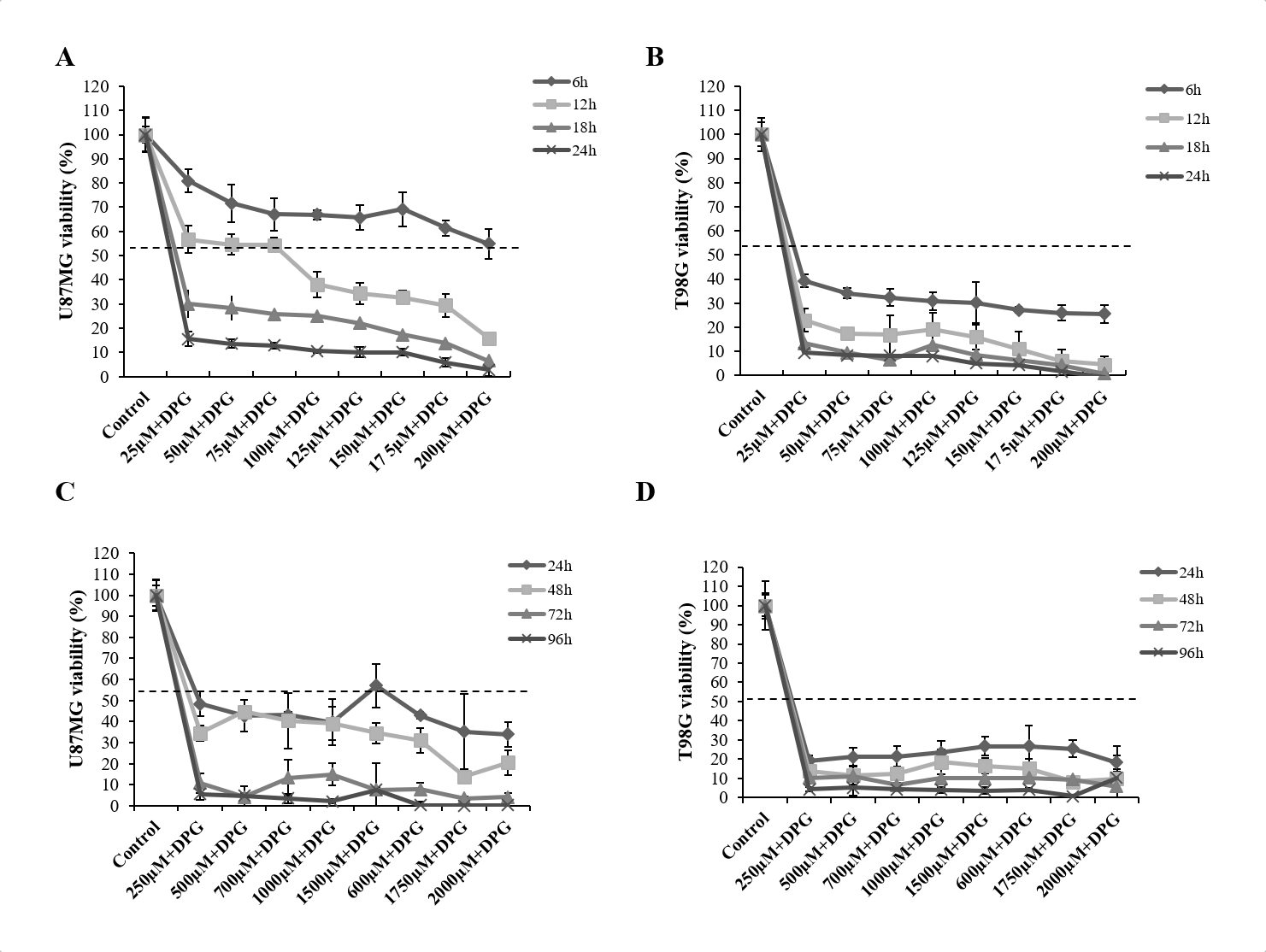

Supplement: FIGURE S2 — Temozolomide (TMZ) in combination with dipotassium glycyrrhizinate (DPG) induces significant dose- and time-dependent reductions of cell viability. Low TMZ concentrations with IC50 DPG was able to induce U87MG (A) and T98G (B) cell viability reduction in incubation times (6, 12, 18, and 24 h) lower than those observed when used alone in a dose- and time-dependent manner. High TMZ concentrations with IC50 DPG was able to induce U87MG (C) and T98G (D) cell viability reduction in incubation times (24, 48, 72, and 96 h) lower than those observed when used alone in a dose- and time-dependent manner. The graphic shows the standard deviation of three independent experiments. Statistics were performed in a two-tailed t-test with P ≤ 0.05. All experiments were performed in triplicate and were repeated at least twice. Graphics are representative of one of three independent experiments. [file Image_2.TIF]

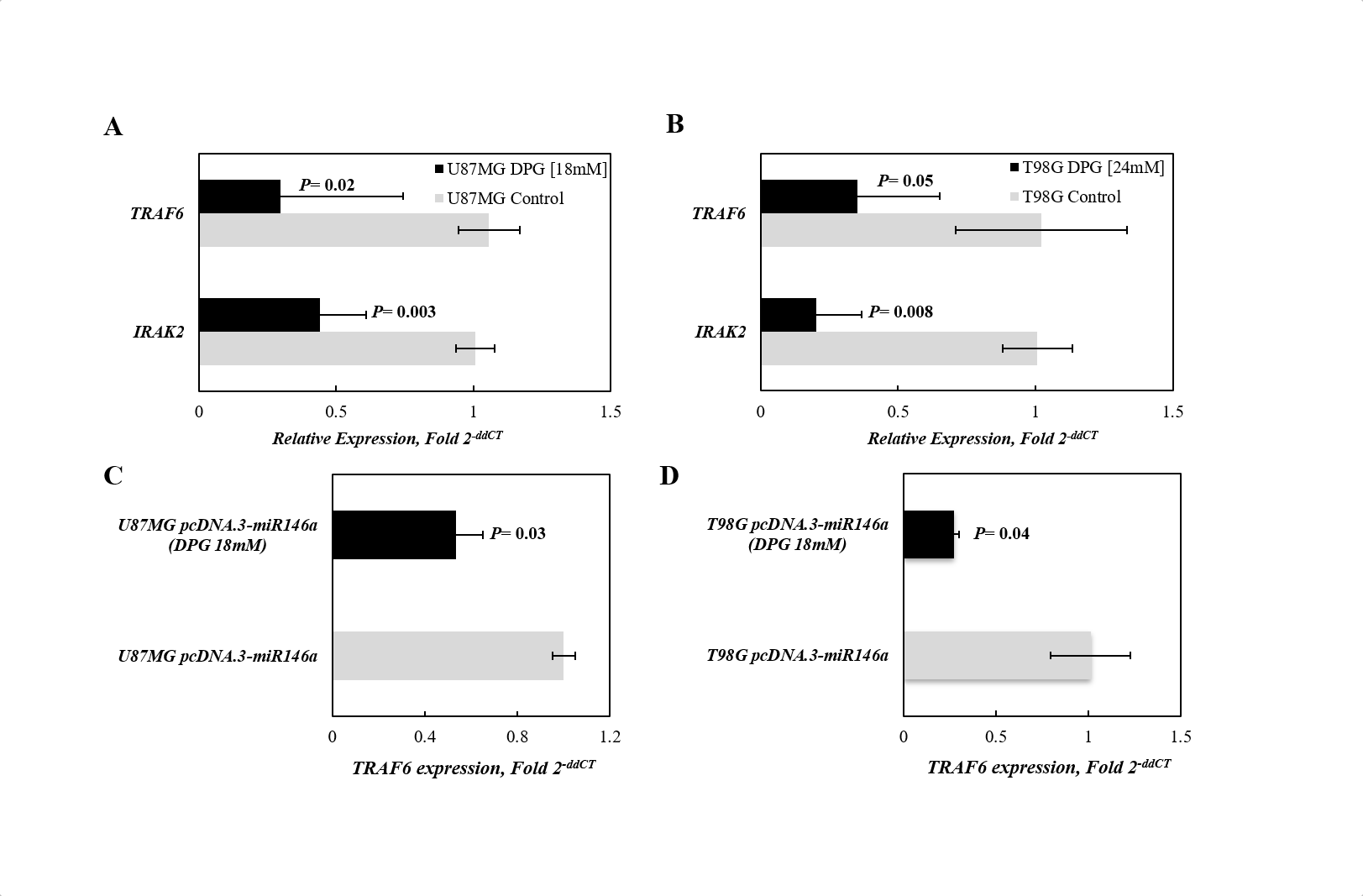

Supplement: FIGURE S3 — Dipotassium glycyrrhizinate (DPG) down-regulates IRAK2 and TRAF6. (A) DPG decreases IRAK2 and TRAF6 mRNA levels in U87MG (P = 0.02 and P = 0.003, respectively) and (B) T98G (P = 0.03 and P = 0.008, respectively) compared to untreated cell lines using 18S reference. DPG decreases IRAK2 and TRAF6 mRNA levels in (C) U87MG-pcDNA3.3-miR146a and (D) T98G-pcDNA3.3-miR146a (P = 0.03 and P = 0.04, respectively) compared to untreated pcDNA3.3-miR146a cells using 18S reference. Data represent means and standard deviations of a representative experiment performed in triplicate. Statistics were performed in a two-tailed t-test with P ≤ 0.05. [file Image_3.TIF]
